# Improving University Students' Mental Health During the COVID-19 Pandemic: Evidence From an Online Counseling Intervention in Italy

**DOI:** 10.3389/fpsyt.2022.886538

**Published:** 2022-05-17

**Authors:** Giovanna Celia, Francesca Tessitore, Elisa Cavicchiolo, Laura Girelli, Pierpaolo Limone, Mauro Cozzolino

**Affiliations:** ^1^Department of Economics, Management and Territory, University of Foggia, Foggia, Italy; ^2^Department of Humanities, Philosophy and Education, University of Salerno, Fisciano, Italy; ^3^Department of Humanities, Literature and Cultural Heritage, University of Foggia, Foggia, Italy

**Keywords:** stress, anxiety, mental distress, time perspective, mind-body therapies, university students, COVID-19 pandemic, Italy

## Abstract

**Background:**

The mental health of university students is significantly affected when faced with public health emergencies and requires specific interventions to help support and prevent any long-lasting effects that the pandemic may have on their mental health status. This study aims to evaluate the impact of an online individual counseling intervention provided by the University of Foggia and carried out during the COVID-19 pandemic on the mental health status of a sample of university students.

**Methods:**

32 Italian undergraduate students took part in a one-group pretest-posttest research design. The data was gathered in two times: before the start of the counseling intervention (T1), positive and negative affect, satisfaction with life, global mental distress, anxiety, stress, and future time perspective were collected, at the end of the counseling intervention (T2), the same dimensions were measured. A one-way repeated measures multivariate analysis of variance (MANOVA) was performed, and single Bonferroni-corrected dependent *t*-tests were conducted on variables showing a significant change over time.

**Results:**

The results showed that positive affect, subjective well-being, and future time perspective increased significantly after the intervention. In contrast, the participants reported significantly lower levels of negative affect, global mental distress, state and trait anxiety, as well as perceived stress over time.

**Conclusions:**

The study demonstrates the promising impact of online counseling intervention and its efficient contribution in promoting the well-being of university students. The results contribute to the ongoing debate concerning the psychological impact of the COVID-19 pandemic on young adults, helping professionals develop more efficient clinical and psychological interventions.

## Introduction

The impact of the COVID-19 pandemic on students' mental health represents a major concern for higher education institutions as well as for the mental health sector worldwide. In response to the pandemic, several governments around the world adopted extraordinary measures and containment efforts (e.g., lockdown) aimed to prevent the high risk of contagion and limit the COVID-19 outbreak. Consequently, universities across the nations rapidly closed, moving to remote methods of teaching and assessment. Although remote learning allowed students to maintain their academic routines (1), research around the world agreed that the COVID-19 pandemic negatively impacted university students' mental health status. Specifically, research showed an increase in depression, anxiety and stress (2–7), post-traumatic stress symptoms (8), somatic symptoms (9), substance use (10), and a worsening of existing mental illnesses (11). Increased suicidal thoughts were also reported (12), with a higher risk for females, and students who already had a history of self-injury and suicidal attempts (7, 13). A general decrease in terms of well-being, with an increase in quantity of sleep but a decrease in terms of quality, was also evidenced (14).

Among the risk and protective factors for mental health, having relatives or friends infected with COVID-19, massive media exposure with low quality and clarity of information, history of psychiatric follow-up, and female gender were considered major risk factors (7, 15–20), whereas, living in urban areas, family income stability, and high social support were protective factors against mental health problems (3, 19, 21).

In Italy, recent clinical assessments confirmed that, during the lockdown, university students experienced high levels of anxiety and depression (22–25), somatic complaints and aggressive behaviors (26), changes in sleep rhythms and symptoms of insomnia (27). However, prospective studies need to be further carried out in order to study the long-term effects of quarantine and the pandemic on mental health (28).

Overall, the scientific community agreed that the mental health of university students is significantly affected when faced with public health emergencies, and requires specific interventions to help, support and prevent any long-lasting effects of the pandemic on their mental health status.

University counseling services can play a fundamental role in offering support to university students. Due to the pandemic, and in order to reduce the high risk of contagion, digital psychological interventions and online counseling services have become even more widespread (29, 30). Literature has already shown that online counseling can be as efficient as face-to-face counseling (31) and that it can be successfully implemented for different mental health issues, such as depression, anxiety, stress, or post-traumatic stress disorders (32, 33). Research has also shown that, during the pandemic, online counseling has been effective and has helped alleviate the psychological suffering of the general population (34). The American Psychological Association also supported this approach by providing guidelines as well as suggesting states to guarantee human welfare and stop the spread of the virus. Considering university students' developmental challenges, the counseling intervention might be particularly suitable for promoting their psychological well-being (35, 36). Harrer et al. (37) showed a significant small-to-moderate effect of internet interventions on university students' mental health in their meta-analysis. Moreover, Bolinski et al. (38) also suggested a promising direction for the effectiveness of e-mental health intervention on academic performances. Recently, Hadler et al. (39) reported that tele-mental health services present the advantage for students to reduce the barriers which they might face in reaching out for professional help, such as the perceived stigma (40, 41).

Research on the implementation of online counseling services for university students during the pandemic is still underdeveloped. Early findings on group interventions confirmed the effectiveness of online counseling compared to face-to-face counseling in decreasing anxiety levels (42, 43).

The current study aims to evaluate the effects of an online individual counseling intervention carried out during the COVID-19 pandemic on the levels of subjective well-being, global distress, emotional health (positive and negative affects and anxiety), and future time perspective in a sample of Italian university students. The variables of well-being, global distress, and emotional health status were chosen following the constructs widely investigated in literature for the mental health assessment of university students. Since the pandemic increased the feeling of uncertainty about the future (44), and the ability to foresee and plan for the future is crucial to young adults' well-being and motivation (45), the dimension of the future time perspective was also considered. We expected the following: a) a decrease in negative affect, perceived stress, global mental distress and anxiety; b) an increase in positive affect, subjective well-being and future time perspective.

## Materials and Methods

### Participants and Procedures

In March 2020, when the COVID-19 pandemic was beginning to spread rapidly in Italy, the Psychological Counseling Service of the University of Foggia started to offer its entire university population (students, teaching staff and non-teaching personnel) a free online service for coping with stress, anxiety, social maladjustment, and negative affect. This counseling service had already been active, offering brief face-to-face psychological interventions that usually consisted of 3–5 sessions. Then, with the spread of the pandemic, it was moved to an online service. To access the psychological counseling service, individuals needed to send a request via email to the institutional account counseling@unifg.it. The service manager would then view and respond to the emails by sending instructions to fill out a form relating to privacy and the authorization of data processing. Once the form was completed, a first welcoming interview was carried out, during which the general state of the individual, their personal and clinical history, previous therapeutic experiences and/or past or present use of drugs and medicine were examined. If psychiatric problems or risks for the person were glimpsed, they would be directed to the local health service.

The present study data were collected in late 2020 and 2021, around the end of the second wave of the COVID-19 pandemic in Italy. In recruiting the sample, the following exclusion criteria were established: a) having a diagnosed psychiatric disorder; b) attending pharmacological therapy for psychiatric disorders. Inclusion criteria were: a) being a student of the University of Foggia; b) not having a diagnosed psychiatric disorder; c) not attending pharmacological therapy for psychiatric disorders.

A total of 56 students were invited to fill out a questionnaire at the beginning and at the end of the counseling intervention. At the time of the second data collection, 24 students could not be contacted for various reasons (e.g., they decided to no longer attend the counseling service) and they were therefore excluded from the study. Thus, the final sample for our study consisted of 32 students (65.6% females; *M*_age_ = 22.88; *SD*_age_ = 2.09). Before starting the research, all the participants were informed about the aims of the study and the pertinent contact modalities and persons. Participation was voluntary and confidentiality was assured before collecting the data, with each participant being assigned an anonymous alphanumeric code. All the students were asked to give their consent to participate in the research and the informed consent application form clearly specified that the participants could withdraw from the study at any time, without having to justify this decision and without incurring any consequences. All the procedures conducted in the study were in accordance with the ethical standards of the Italian Association of Psychology (AIP), as well as the 1964 Helsinki declaration and its subsequent amendments specifying the ethical principles that ensure the protection of individuals participating in medical research.

The study design was based on a one-group pretest-posttest research design. The participants were asked to fill out an on-line questionnaire sent via Google Form before the start and at the end of the counseling intervention, after a total of five counseling meetings. Therefore, the data was gathered in two phases: before the beginning of the counseling intervention, Time 1 (T1), the measures of positive and negative affect, satisfaction with life, global mental distress, anxiety, stress, and future time perspective were administered; at the end of the counseling intervention, Time 2 (T2), the same measures were administered.

The intervention provided to students by the Psychological Counseling Service of the University of Foggia works effectively and multidimensionally on the individual, also including “non-pathological” realities (42). The psychological interventions proposed are part of an integrated mind-body approach with strategic orientation (46–50). This approach arises from the need to convey, in a coherent and organized way, the contributions of several schools of thought in an operational, flexible, effective, efficient model (51). The counseling intervention is short with a maximum duration of 5 interviews. It is focal because it works in a targeted manner on the reported problems and is also configured as a strategic process that aims to consolidate the new equilibrium reached. Broadly, during the counseling sessions, the therapist, together with the student, identifies the problems to be solved, establishes the objectives, plans the interventions to achieve these objectives and, at the end of the course, examines the results to see if the intervention has been successful. The psychological counseling provided was organized in four phases (46, 52–56): (1) definition of the problem in concrete terms; (2) analysis of the solutions attempted to solve the problem; (3) concrete definition of the change to be made; (4) formulation and implementation of a change plan.

The counseling path includes the following techniques (53):

- empathic listening in order to let the individual speak openly, constructing and verifying their own hypotheses through their narratives, inserting themselves, mostly with open questions, which avoid influencing the individual's answers and preserve maximum freedom of expression.
- Reformulation of the student's verbal and non-verbal communication to implement a restructuring of the narrative, referring new elements and meanings to the individual, derived from their own hypotheses (55).
- Reframing and feedback to guide the student toward change.

During the entire counseling process, the therapist:

- allows the individual to experiment with the solutions built in the interview, while still having a “protected” space available to process the effects;
- supports and consolidates the new ways of interacting with the student, putting them in relation to the old schemes;
- outline the problematic issues faced by the student, relating them to the new strategies implemented;
- acknowledges and positively emphasizes the commitment of the individual in achieving the agreed objectives.

### Measures

All the instruments were administered in Italian, the participant's first language. All the measures were already available in Italian, so it was not necessary to translate them.

In order to measure positive and negative affect, the Positive and Negative Affect Schedule (PANAS) (57, 58) was used. The PANAS is a well-known scale that measures the most general dimension of affective experience, and it includes two 10-item mood scales. The Positive Affect scale reflects a pleasurable engagement with the environment and measures the extent to which a person feels determined, excited, or enthusiastic; instead, the Negative Affect scale reflects a variety of unpleasant mood states such as feeling upset, scared or nervous. The participants were asked to indicate how often over the past week they had experienced the different feelings and emotions described in each item using a scale ranging from 1 (“very slightly”) to 5 (“extremely”). Several research has supported the excellent psychometric proprieties of the PANAS [e.g., (59, 60)], and in the Italian context, there is evidence to support its reliability and validity (57). In the present study the alpha coefficient was 0.91 (at T1) and 0.90 (at T2) for PA, and 0.92 (at T1) and 0.94 (at T2) for NA.

We used The Satisfaction With Life Scale (SWLS) (61) as a measure of individuals' global satisfaction with life. The scale reflects the judgmental component of subjective well-being and includes five items (sample item: “In most ways my life is close to my ideal”). Students were asked to indicate how much they agree or disagree with each of these five items using a 5-point scale that ranges from 1 (“strongly disagree”) to 5 (“strongly agree”). The scale has shown to be a valid and reliable measure of life satisfaction [e.g., (62, 63)]. The psychometric properties of the scale have also been supported in the Italian context (64). The alpha coefficients for T1 and T2 were 0.80 and 0.83, respectively.

In the present study, The Young Person's Clinical Outcomes in Routine Evaluation (YP-CORE) (65, 66) was also included. The YP-CORE is a brief scale, especially designed for young people attending counseling or therapy, which measures global mental distress. It comprises 10 self-report items which cover the psychological domains of well-being (sample item: “My problems have felt like too much for me”), risk to self (sample item: “I've thought about hurting myself”), symptoms/problems (sample item: “I've felt unhappy”) and functioning (sample item: “I've felt able to cope when things go wrong”). The students rated how often over the last week they had felt in the way described by each item, by using a 5-point scale that ranges from 1 (“not at all”) to 5 (“most or all of the time”). The psychometric properties of the YP-CORE have been investigated in clinical as well as in nonclinical samples [e.g., (67, 68)] and there is evidence of its good psychometric properties in different countries [e.g., (69, 70)], as well as in the Italian context (71). The alpha coefficient for the current study was 0.74 at T1 and 0.84 at T2.

Anxiety was assessed by the Y form of the State-Trait Anxiety Inventory (STAI-Y) (72), which is made of two separate self-report scales for measuring state and trait anxiety. The state anxiety was measured by the S-Anxiety scale (STAI Form Y-1) which is made of 20 items that evaluate how the participants feel “right now, at this moment” (sample items: “I am worried”, “I feel calm”). The students were therefore asked to rate the extent to which they were feeling tense, nervous or worried at the moment, on a scale ranging from 1 (“not at all”) to 4 (“very much so”). The trait anxiety was instead measured by the T-Anxiety scale (STAI Form Y-2) which consists of 20 items that assess how the respondents generally feel anxious and refers to relatively stable aspects (sample items: “I worry too much over things that really don't matter”, “I am a steady person”). In this case the students rated the extent to which they commonly feel tense, nervous or worried, on a scale ranging from 1 (“almost never”) to 4 (“almost always”). The STAI is one of the most used measures of state and trait non-disorder-specific anxiety (73–76) and it has shown excellent psychometric properties [e.g., (77–79)], including in the Italian context (80). In the current study, internal reliability of S-Anxiety was 0.94 at T1 and 0.95 at T2, while it was 0.89 (at T1) and 0.92 (at T2) for T-Anxiety.

Stress was assessed by means of the single-item Distress Thermometer (DT) (81). Students were asked to indicate the stress they had perceived over the last week on an 11-point scale, ranging from 0 (no distress) to 10 (extreme distress). The DT has proven to be a sensitive tool to assess the construct of psychosocial distress (82), and it has also proved to be effective in the Italian context (83–85).

To measure the future time perspective, we used the 9-item scale which measures the future temporal frame of the Zimbardo Time Perspective Inventory Short Form (ZTPI-short version) (86, 87). This scale reflects a future-oriented temporal frame and describes individuals as having a strong sense of purpose for the future (sample item: “When I want to achieve something, I set goals and consider specific means for reaching those goals”). Replies for each item were chosen from a 5-point scale ranging from 1 (“strongly disagree”) to 5 (“strongly agree”). Several reviews of time perspective instruments have found the ZTPI to be a valid and reliable measure [e.g., (88)]. The Italian version of the future scale has good psychometric properties (87). In the present study the alpha coefficient was 0.71 at T1 and 0.68 at T2.

### Analysis

The analysis was conducted by using the IBM SPSS Statistics for Windows, version 23 (IBM Corp., Armonk, N.Y., USA). Bivariate correlations and descriptive statistics were computed for all the study variables at both time points. A set of independent *t*-tests were performed on the study variables to detect possible differences between those who remained and dropped out with respect to the study variables at T1. A one-way repeated measures multivariate analysis of variance (MANOVA) with time (T1 and T2) as the grouping factor was performed to examine changes in students' subjective well-being, global distress, emotional health (positive and negative affects and anxiety), and future time perspective. Finally, in order to investigate where significant differences occurred before and after the intervention, we performed Bonferroni-corrected dependent *t*-tests on variables showing a significant change over time.

## Results

Descriptive statistics of all the study variables for the two time points are presented in Table 1 while bivariate correlations are shown in Table 2.

**Table 1:** Descriptive statistics of the study variables.

	**range**	***M***	***SD***	***Min***	***Max***
**Pretest variables**					
Positive affect (PA)	1–5	2.34	0.82	1.2	4.1
Negative affect (NA)	1–5	3.12	0.93	1.6	5
Subjective well-being (SWLS)	1–5	2.59	0.85	1	4.4
Global mental distress (YP-CORE)	1–5	3.19	0.65	1.7	4.3
State anxiety (S-Anxiety)	1–4	2.72	0.62	1.6	3.9
Trait anxiety (T-Anxiety)	1–4	2.87	0.46	2.3	3.9
Perceived stress (DT)	0–10	7.16	2.02	1	10
Future time perspective (FTP)	1–5	3.25	0.51	2	4.2
**Posttest variables**					
Positive affect (PA)	1–5	2.86	0.77	1.3	4.1
Negative affect (NA)	1–5	2.52	0.93	1.1	4.4
Subjective well-being (SWLS)	1–5	2.89	0.81	1.6	4.8
Global mental distress (YP-CORE)	1–5	2.57	0.79	1.4	4.3
State anxiety (S-Anxiety)	1–4	2.32	0.67	1.2	3.6
Trait anxiety (T-Anxiety)	1–4	2.65	0.54	1.6	3.5
Perceived stress (DT)	0–10	5.91	2.18	2	10
Future time perspective (FTP)	1–5	3.43	0.47	2.6	4.6

*Note: M, mean; SD, standard deviation; Min, minimum; Max, maximum*.

**Table 2:** Bivariate correlations between study variables.

**Pretest variables**	**1**	**2**	**3**	**4**	**5**	**6**	**7**	**8**
Positive affect (PA)	-							
Negative affect (NA)	0.22	-						
Subjective well-being (SWLS)	0.42^**^	−0.33^*^	-					
Global mental distress (YP-CORE)	−0.58^***^	0.62^***^	−0.52^***^	-				
State anxiety (S-Anxiety)	−0.32^*^	0.70^***^	−0.26	0.62^***^	-			
Trait anxiety (T-Anxiety)	−0.48^***^	0.63^***^	−0.53^***^	0.76^***^	0.68^***^	-		
Perceived stress (DT)	−0.10	0.58^***^	−0.17	0.47^***^	0.53^***^	0.48^***^	-	
Future time perspective (FTP)	0.68^***^	−0.27^*^	0.49^***^	−0.53^***^	−0.26	−0.48^***^	−0.01	-
**Posttest variables**								
Positive affect (PA)	-							
Negative affect (NA)	−0.49^**^	-						
Subjective well-being (SWLS)	0.43^*^	−0.27	-					
Global mental distress (YP-CORE)	−0.70^***^	0.68^***^	−0.42^*^	-				
State anxiety (S-Anxiety)	−0.47^**^	0.61^***^	−0.50^**^	0.78^***^	-			
Trait anxiety (T-Anxiety)	−0.68^***^	0.61^***^	−0.63^***^	0.82^***^	0.74^***^	-		
Perceived stress (DT)	−0.30	0.44^*^	−0.24	0.58^***^	0.57^***^	0.51^**^	-	
Future time perspective (FTP)	0.62^***^	−0.25	0.53^**^	−0.33	−0.28	−0.56^***^	−0.17	-

* *p < 0.05;*

** *p < 0.01;*

*** *p < 0.001*.

No significant differences were detected between the two groups (remainers and dropouts) at T1 either for negative affect [*t*_(54)_ = 1.84; *p* =0.07], subjective well-being [*t*_(54)_ = −1.12; *p* = 0.27], global mental distress [*t*_(54)_ = 1.77; *p* = 0.08], state anxiety [*t*_(54)_ = 0.80; *p* = 0.43], trait anxiety [*t*_(54)_ = 1.43; *p* = 0.16], or stress [*t*_(54)_ = 0.88; *p* = 0.38]. We found a statistically significant difference between the two groups for positive affect [*t*_(54)_ = −2.11; *p* = 0.04], with the dropouts reporting higher levels of positive emotions than those who participated in the counseling sessions (dropouts: *M* = 2.83; *SD* = 0.94; remainers: *M* = 2.34 = *SD* = 0.82). Finally, there was a statistically significant difference between remainers and dropouts with regard to the future time perspective [*t*_(54)_ = −2.72; *p* = 0.01), with the dropouts indicating higher levels of future time perspective compared to the remainers (dropouts: *M* = 3.65; *SD* = 0.59; remainers: *M* = 3.25; *SD* = 0.51).

The results of the repeated measures MANOVA analysis indicated significant changes in the variables considered before and after the intervention [Wilks's Λ =0.45, *F*_(8, 24)_ = 3.66, *p* = 0.006]. The results of the Bonferroni-corrected dependent *t*-tests showed that positive affect [*t*_(31)_ = −3.52, *p* = 0.001, Cohen's *d* = −0.62], subjective well-being [*t*_(31)_ = −3.28, *p* = 0.001, Cohen's *d* = −0.58] and future time perspective [*t*_(31)_ = −3.22, *p* = 0.003, Cohen's *d* = −0.57) increased significantly across the two time points. By contrast, the participants reported significantly lower levels of negative affect [*t*_(31)_ = 3.73, *p* < 0.001, Cohen's *d* = 0.66], global mental distress [*t*_(31)_ = 4.54, *p* < 0.001, Cohen's *d* =0.80], and state [*t*_(31)_ = 3.36, *p* = 0.002, Cohen's *d* = 0.59] and trait anxiety [*t*_(31)_ = 3.24, *p* = 0.003, Cohen's *d* = 0.57], as well as perceived stress [*t*_(31)_ = 3.75, *p* < 0.001, Cohen's *d* = 0.66] after the intervention. All the variables showed differences that represented a medium effect size, except for the one detected for global mental distress which represented a large effect size.

## Discussion

Young people are exposed to different specific threats to their mental health (89, 90) and the present study evaluated the effectiveness of an online individual counseling intervention on a sample of 32 Italian undergraduate students during the COVID-19 pandemic. Using an uncontrolled pretest/post-test design, the effects of a short-term individual online counseling intervention on the perception of stress, global distress, subjective well-being, emotional health status (positive and negative affects and anxiety), and future time perspective was investigated by comparing the levels of these psychological dimensions at the beginning (T1) and at the end (T2) of the intervention.

In line with existing evidence on the effectiveness of university face-to-face counseling (35, 36), the results confirmed our hypothesis showing a significant decrease of global mental distress and perceived stress after the online intervention. The effectiveness of the online counseling intervention in reducing the levels of anxiety, both state and trait, and in increasing the individual's subjective well-being was also confirmed. With regards to the emotional health status, our hypotheses were also confirmed since the results showed a growth of positive affect and a reduction of negative one at the T2 of the intervention. These results are consistent with existing studies on the efficacy of online group counseling in promoting well-being and increasing the emotional health of undergraduate students during the pandemic (42, 43).

The online counseling intervention also produced an increase in student's future time perspective (FTP), that is, a higher tendency for them to think about the future in terms of goals to be achieved and tasks to be done. The present study is the first to have investigated this psychological dimension on young adults during the COVID-19 pandemic, even though research on time perspective and future orientation is crucial to understanding how people are dealing with this pandemic, especially among young people for which future aspirations are so salient (91). Several studies demonstrated that time perspective is an important psychological variable associated with many areas of human functioning (i.e., well-being, health behaviors, risky behaviors) (92–94). More recently, O'Neill et al. (95) also found that FTP is related with psychological resilience, intended as a core component of mental health. Particularly for young adults, planning for the future represents an important developmental task that becomes fundamental in order to orient and guide the students in their career decision-making (96–98). Therefore, this result shed light on the potential of online psychological counseling in increasing university student's planning for the future, restoring the fragmentation in their views toward the future and the sense of timelessness that the COVID-19 pandemic produced.

In conclusion, the results suggested that a short-term online individual psychological counseling intervention represents an effective and incisive way to face the emerging distress and discomfort within university environments. Taking into consideration the negative impact of the COVID-19 pandemic on student's mental health, we strongly encourage universities to improve the planning of online counseling services in order to facilitate the creation of supportive and meaningful spaces that are able to prevent student's mental health problems, promote their subjective well-being, and elaborate the emotional turmoil provoked by the COVID-19 pandemic.

### Limitations

Although our results indicate that counseling interventions can be a promising mental health promoting action, the study design is not free from limitations. Firstly, we excluded students who presented psychiatric disorders or who were receiving pharmacological treatment for psychiatric disorders, however, this exclusion criteria was mainly based on information reported by the students themselves and by the clinical evaluation of the therapist. Secondly, the lack of a control group did not allow us to draw definitive conclusions regarding the effects of the counseling intervention and caution must be made when interpreting the presented results as other factors besides the intervention may have played a role in the changes observed. The intervention described in the present study was part of the normally routine care of the counseling center, therefore no control group was possible for ethical reasons. Moreover, in order to sustain the students' mental health during the disruptive period of the second wave of the COVID-19 pandemic in Italy, the management of the counseling service decided not to activate a waiting list and to welcome all students who requested the online service. Despite this limitation, our results appeared to be in line with previous studies which have proved the efficacy of university counseling interventions in reducing mental distress and improving psychological well-being (e.g., 35, 36, 42, 43). Therefore, we believe that our findings provide the basis on which to build future research studies, which could involve more participants, multiple sites, and a control group. Thirdly, the number of participants resulted in a small sample for the analysis. Finally, the attrition rate was quite high. This is quite common in clinical intervention (99) and can also depend on whether the intervention is online (100). Future studies can promote greater levels of engagement and intrinsic motivation in students to reduce this high rate. Future research also needs to investigate the effect of online counseling intervention on other variables, such as academic performances and career oriented decision-making as well as deepen the effectiveness of different techniques and their specific impact on mind-body processes (100–105). Moreover, future studies should enhance the implementation of qualitative and multilevel investigations in this field, taking into consideration its value in shedding light on the affective processes and subjective meanings of the experiences in different populations (106–112).

## Conclusion

The study enriched the still limited field of studies about the effectiveness of online counseling interventions and their impact on the mental health status of university students during the pandemic. It contributed to the ongoing debate concerning the psychological impact of the COVID-19 pandemic on young adults, demonstrating the promising impact of online counseling intervention and its efficient contribution in promoting the well-being of university students. Taking into consideration the unknown long-lasting effects of the pandemic on mental health, the study called for enabling support environments that allow university students to meaningfully participate in transformative and healthy opportunities.

## Data Availability Statement

The raw data supporting the conclusions of this article will be made available by the authors, without undue reservation.

## Ethics Statement

The studies involving human participants were reviewed and approved by the ethical standards of the Italian Association of Psychology (AIP), as well as the 1964 Helsinki declaration and its subsequent amendments specifying the ethical principles that ensure the protection of individuals participating in medical research. The patients/participants provided their written informed consent to participate in this study.

## Author Contributions

GC and MC developed the theoretical framework of the present study, designed the study, and developed the methodological approach. EC and LG performed all the analyses and designed tables and figures. FT led the literature search and interpretation of data. GC, MC, and PL critically revised the manuscript. MC contributed to the scientific supervision of the whole work. All authors made a substantial contribution to the work, read, and approved the final version of the work.

## Funding

This study was funded by the University of Foggia (TASSE TFA_SOSTEGNO 19/20_Traetta).

## Conflict of Interest

The authors declare that the research was conducted in the absence of any commercial or financial relationships that could be construed as a potential conflict of interest.

## Publisher's Note

All claims expressed in this article are solely those of the authors and do not necessarily represent those of their affiliated organizations, or those of the publisher, the editors and the reviewers. Any product that may be evaluated in this article, or claim that may be made by its manufacturer, is not guaranteed or endorsed by the publisher.
